# Optically Activated Superconductivity in MgB_2_ via Electroluminescent GaP Inhomogeneous Phase

**DOI:** 10.3390/ma19071456

**Published:** 2026-04-05

**Authors:** Yao Qi, Duo Chen, Qingyu Hai, Xiaoyan Li, Xiaopeng Zhao

**Affiliations:** Smart Materials Laboratory, Department of Applied Physics, Northwestern Polytechnical University, Xi’an 710129, China; qiyao@mail.nwpu.edu.cn (Y.Q.); chenduo@mail.nwpu.edu.cn (D.C.); haiqingyu@mail.nwpu.edu.cn (Q.H.); lixiaoyan0521@mail.nwpu.edu.cn (X.L.)

**Keywords:** MgB_2_, electroluminescent inhomogeneous phase, critical current density, flux pinning, electron–phonon coupling, multi-quantum-well particles, smart meta-superconductors

## Abstract

Experimental results suggest a feasible strategy for tuning the superconducting properties of MgB_2_ through the incorporation of an electroluminescent inhomogeneous phase. By introducing GaP electroluminescent inhomogeneous phases into MgB_2_, the effects of emission intensity variation on the sample structure, superconducting transition temperature, electrical transport behavior, and magnetic properties were systematically investigated. The results show that, at a fixed GaP addition level, the superconducting transition temperature *T_c_* increases steadily from 38.2 K to 39.6 K with increasing emission intensity of the inhomogeneous phase, corresponding to a maximum enhancement of approximately 1.4 K. Meanwhile, the zero-resistance temperature shifts upward synchronously, indicating that the entire superconducting transition region moves toward higher temperatures. Raman measurements show that the peak position and linewidth of the *E_2g_* phonon mode evolve systematically with emission intensity, while the electron–phonon coupling parameter *λ* exhibits a trend consistent with that of *T_c_*. In addition, the nanoscale dispersed distribution of the GaP inhomogeneous phase, together with the interface/defect structures it introduces, appears to promote sample densification and enhance flux pinning, resulting in an increase in the critical current density *J_c_* by approximately 69% at 20 K in self-field and an enhancement of the irreversibility field *H_irr_* by about 31.5%. These results suggest that, beyond the effect of static inhomogeneous-phase incorporation, the luminescence-activated state under bias excitation is likely involved in modulating the superconducting response of MgB_2_. This work provides a new experimental perspective for synergistically regulating the properties of conventional superconductors through the combined effects of inhomogeneous phases and excited states.

## 1. Introduction

Layered structural systems play a central role in modern superconductivity research. From moiré-band engineering in magic-angle graphene systems [1,2] to the layered coordination geometries in nickel-based and iron-based superconductors [3,4,5,6], and to the alternating Mg and B layers forming the two-dimensional framework of MgB_2_, the electronic properties and quantum behavior of these materials are strongly governed by the band structures and vibrational modes dictated by their layered crystal architectures. In the MgB_2_ system in particular, its characteristic two-band superconductivity and strong electron–phonon coupling originate directly from the unique soft-mode behavior of the *E_2g_* phonon associated with the B-B σ-bonding network [7,8].

Among various superconducting systems, MgB_2_, as a representative electron–phonon-coupled superconductor, has attracted extensive attention since its discovery because of its high critical temperature of 39 K, weak anisotropy, low cost, and excellent processability, which together make it highly suitable for superconducting magnets, power devices, and quantum applications [9]. However, the superconducting properties of MgB_2_ are highly sensitive to the electron–phonon coupling strength and grain connectivity. Conventional approaches such as chemical doping, nanoparticle inclusion, and microstructural engineering may improve certain performance metrics, yet they commonly suffer from issues including *T_c_* suppression caused by lattice distortion, chemical instability, interfacial side reactions, and enhanced grain-boundary scattering [10,11,12,13,14,15]. Therefore, enhancing electron–phonon coupling and improving superconducting performance without perturbing the intrinsic lattice structure remains a critical scientific challenge in MgB_2_ research.

In recent years, interfacial electromagnetic excitation, plasmonic near-field effects, and light-phonon cooperative interactions have demonstrated the capability to modulate electronic structures and coupling mechanisms in condensed-matter systems, offering a new perspective beyond conventional superconductivity-control pathways [16,17,18,19,20,21]. Under optical near-field excitation, lattice vibrations can be dynamically tuned, and electron–phonon interactions may be enhanced by modifying the local electromagnetic environment [22,23,24,25]. However, such light-field-based modulation typically relies on pulsed light sources or complex external-field conditions, making it difficult to implement directly in bulk superconductors [26]. The ability to create internally embedded, stable, and continuously excitable luminescent interfaces capable of generating localized optical fields would thus open new material systems and physical mechanisms for superconductivity regulation.

Guided by the design principles of intelligent metamaterials, our previous work proposed a strategy termed “electroluminescent inhomogeneous phase-enhanced superconductivity.” By introducing electroluminescent inhomogeneous phases into MgB_2_ or cuprate superconductors such as Bi(Pb)SrCaCuO and applying bias currents during electrical transport measurements, we demonstrated that the resulting interfacial electroluminescence and localized electromagnetic near-fields can construct smart meta-superconductors (SMSCs) [27,28,29,30,31,32,33,34,35,36]. We employed several types of luminescent inhomogeneous phases, including Y_2_O_3_:Eu^3+^ + Ag particles and GaN p-n junctions, and experimentally verified that current-induced interfacial emission can enhance superconducting performance across different superconducting systems. We further proposed a physical model in which interfacial plasmons and their evanescent electromagnetic fields strengthen electron pairing [30]. In our previous work, we systematically investigated the effects of the inhomogeneous phase particle size and addition concentration on the superconducting properties, identifying 0.5 wt.% as the optimal addition level that balances the interfacial excitation effect with the connectivity of MgB_2_ [32,33]. Therefore, in the present study, the content of the GaP inhomogeneous phase was fixed at 0.5 wt.% to minimize the interference arising from compositional or concentration variables, allowing the investigation to focus specifically on the influence of “tunable electroluminescence intensity” on the superconducting performance. However, the electroluminescent inhomogeneous phases used thus far exhibit limitations in emission intensity, directionality, and crystalline symmetry. Notably, the systematic influence of increased emission intensity on superconducting behavior has yet to be experimentally elucidated.

Building on these previous studies, the present work introduces isotropic GaP electroluminescent nanoparticles whose emission intensity can be tuned through periodic structural modulation, and systematically investigates how variations in the emission intensity of the electroluminescent inhomogeneous phase affect the structure, superconducting transition temperature, electrical transport behavior, and magnetic hysteresis of MgB_2_. The possible role of interfacial optical fields in modulating electron–phonon coupling is also discussed. The results show that, with increasing emission intensity, the *E_2g_* phonon mode, *T_c_*, and magnetic properties of the samples exhibit systematic evolution. In particular, the enhancement of *T_c_* is consistent with the changes observed in the Raman features, supporting the interpretation that, beyond the effect of static inhomogeneous-phase incorporation, the interfacial optical field may participate in the modulation of electron–phonon interactions. At the same time, the nanoscale dispersed distribution of the GaP inhomogeneous phase, together with the fine-scale defects/interfaces introduced by it, can serve as effective pinning centers and promote densification, thereby improving *J_c_* and the pinning force and enabling the synergistic optimization of key superconducting parameters. These results suggest that electroluminescent inhomogeneous phases may serve as a promising platform for investigating the relationship between optically excited interfacial states and superconducting response, although the specific microscopic mechanism still requires further rigorous experimental and theoretical verification.

## 2. Materials and Methods

### 2.1. Preparation of GaP Electroluminescent Particles

GaP electroluminescent inhomogeneous phases were synthesized via a hot-injection method. The precursor system was prepared by dissolving gallium acetylacetonate, tris(trimethylsilyl)phosphine, zinc acetylacetonate, tellurium powder, and indium chloride in octadecene (ODE) according to predetermined molar ratios. The reaction was carried out in a three-neck flask under an inert atmosphere. After heating the mixture to 300 °C, tris(trimethylsilyl)phosphine and trioctylphosphine (TOP) were swiftly injected to initiate nucleation and the subsequent growth of multilayer core–shell structures. By controlling the injection sequence and the dwelling time, two classes of multilayer core–shell units, namely [GaP:Zn/GaP]_×m_/[GaInP/GaP]_×n_ and [GaP:Te/GaP]_×m_/[GaInP/GaP]_×n_, were constructed, and these units eventually formed nanoparticles with diameters of approximately 7 nm during the later stage of growth.

Upon completion, the reaction mixture was naturally cooled to room temperature and washed several times with ethanol via centrifugation to remove unreacted precursors. The resulting wet product was dispersed and dried to form a gel-like precursor film, which was subsequently subjected to high-temperature treatment in a tubular furnace under flowing Ar to eliminate organic residues and promote the aggregation of nanocrystals into electroluminescent particles. The final product was ball-milled to narrow the particle-size distribution to 400 nm, yielding GaP electroluminescent inhomogeneous phase particles suitable for composite fabrication.

To obtain GaP inhomogeneous phases with varying intrinsic luminescence intensities, we tuned the number of repetitions of the multilayer core–shell units (m, n) to modify carrier recombination pathways and radiative efficiency, thereby producing a series of particles with different emission efficiencies. After high-temperature treatment and ball milling, each batch of particles was excited under a constant bias current of *I_bias_* = 100 mA using a custom-built testing fixture, and the electroluminescence (EL) spectra were collected via a fiber-optic spectrometer. To enable a direct comparison of the luminescence capability of different samples, the collected electroluminescence spectra were background-subtracted and integrated over the 500–750 nm range under identical measurement conditions, including the same bias condition, optical path, and detection settings, with a fixed integration time of 3 ms. The resulting integrated signal was defined as the relative electroluminescence (EL) intensity of the sample (a.u.). Accordingly, the GaP batches used for composite fabrication were denoted as EL = 0, 1000, 2200, 3960, 4900, 5800, and 6600.

### 2.2. Preparation of MgB_2_ and MgB_2_-GaP Composite Samples

Both the MgB_2_ matrix and MgB_2_-GaP composite samples were prepared using a non-in situ sintering process. The selected GaP content (0.5 wt.%) was based on prior optimization experiments. All weighing, mixing, and transfer operations were performed inside an Ar-filled glovebox with O_2_ and H_2_O levels < 0.1 ppm to avoid oxidation of MgB_2_. Commercial MgB_2_ powder (99.9%, Alfa Aesar) and the GaP inhomogeneous phase particles were separately dispersed in ethanol and ultrasonicated for 20 min to prevent agglomeration. Each dispersion was then magnetically stirred for 20 min to form stable suspensions. The GaP suspension was subsequently added dropwise into the MgB_2_ suspension and stirred for an additional 20 min to ensure uniform dispersion. The mixed suspension was dried under vacuum at 60 °C for 4 h to obtain black composite powder, which was ground for 1 h in an agate mortar to further improve homogeneity. The composite powder was cold-pressed into cylindrical pellets (diameter: 11 mm; height: 1.2 mm) under a pressure of 14 MPa and sealed in tantalum containers to prevent contamination during sintering. Sintering was conducted in a tubular furnace under ultra-high-purity Ar: the pellets were heated to 850 °C at a rate of 5 °C min^−1^ and held for 10 min, followed by cooling to 650 °C at −5 °C min^−1^ and annealing for 1 h. The samples were finally furnace-cooled to room temperature. All samples contained a fixed GaP loading (0.5 wt.%) and were labeled S_1_–S_8_ according to the electroluminescence intensity of the GaP inhomogeneous phases used, enabling systematic investigation of the influence of luminescence strength on the superconducting properties of MgB_2_.

### 2.3. Characterization Methods

The crystal structure of the samples was analyzed using an X-ray diffractometer (D8 Advance, Bruker AXS GmbH, Karlsruhe, Germany; Cu Kα radiation, *λ* = 1.5418 Å). Microstructural morphology was examined by scanning electron microscopy (SEM, Verios G4, Thermo Fisher Scientific, Waltham, MA, USA), and elemental distribution was obtained via energy-dispersive X-ray spectroscopy (EDS, NORAN System 7, Thermo Fisher Scientific, Madison, WI, USA). Electrical transport properties were measured using a standard four-probe method, with low-temperature conditions provided by a closed-cycle cryogenic system (Advanced Research Systems, Macungie, PA, USA; base temperature ≈ 10 K). Electrical transport measurements were performed in the Delta mode using a Keithley 6221 current source and a Keithley 2182A nanovoltmeter (Keithley Instruments, Cleveland, OH, USA). In this mode, the voltage signals under positive and negative current directions are acquired alternately, which effectively eliminates thermoelectric offsets and significantly suppresses background drift and low-frequency noise, thereby improving the stability of low-resistance measurements. The 100 mA bias current used in this study was applied not only to obtain the resistance signal of the sample, but also to simultaneously excite the luminescence of the GaP inhomogeneous phase. Magnetic measurements were performed on a physical property measurement system (CFMS-14T, Cryogenic Ltd, London, UK). The magnetic critical current density *J_c_* was calculated from the magnetic hysteresis loops (*M* − *H*) using the Bean model:

$$J_{c}=\frac{20\Delta M}{a^{2}bh(1-\frac{a}{3b})}\;(with\;b>a)$$

where Δ*M* is the width of the *M* − *H* loop (emu), and *a*, *b*, and *h* represent the width, length, and thickness of the sample, respectively. Samples used for magnetic measurements were cut from the central region of the cylindrical pellets, with typical dimensions of 1.5 mm × 2 mm × 1 mm. During measurement, the ab-plane was oriented perpendicular to the external magnetic field, and the field sweep rate was set to 50 mT s^−1^. The critical current density *J_c_* was derived from the magnetic hysteresis loops based on the Bean model. The associated uncertainty mainly originates from the measurement reproducibility of the magnetic moment difference Δ*M*, the errors in determining the sample geometry and volume, and the propagation of these uncertainties in the model calculation. In this study, the irreversibility field *H_irr_* was defined as the magnetic field at which *J_c_* drops to 10^2^ A·cm^−2^. Raman spectra were collected on a WITec Alpha300R confocal micro-Raman system using a TEM_00_ 532 nm laser as the excitation source and a 600 g mm^−1^ grating for spectral acquisition.

### 2.4. Calculation of Electron–Phonon Coupling Constant (λ)

The electron–phonon coupling constant *λ* was determined from the characteristic phonon frequencies obtained through three-peak Gaussian fitting of the Raman spectra. Raman spectra in the 200–1000 cm^−1^ range were fitted with three Gaussian components corresponding to the low-frequency acoustic mode, the mid-frequency *E_2g_* mode, and the high-frequency second-order/interface-related vibrational mode. The peak positions *ω_i_* and peak areas *A_i_* extracted from the fitting were used to calculate the weight of each phonon mode in the overall coupling process:

$$w_{i}=\frac{A_{i}}{\sum_{j}A_{j}}$$

The logarithmic averaged phonon frequency was then obtained according to the Allen–Dynes approximation:

$$\langle \omega_{log}\rangle =exp\left(\sum_{i}\omega_{i}ln\omega_{i}\right)$$

The relationship between the superconducting transition temperature *T_c_* and the electron–phonon coupling constant *λ* was determined using the Allen–Dynes modified McMillan formula:

$$T_{c}=\frac{\langle \omega_{log}\rangle }{1.2}exp\left(-\frac{1.04(1+\lambda )}{\lambda -\mu^{*}(1+0.62\lambda )}\right)$$

where μ* = 0.13 is the commonly used Coulomb pseudopotential. By substituting the experimentally measured *T_c_* and ⟨*ω_log_*⟩ into the above equation, *λ* can be obtained. This method relies on an indirect extraction of *λ* from the characteristic frequencies obtained by Raman fitting together with the experimental *T_c_*, and thus should be considered semi-quantitative in nature. The resulting *λ* values are therefore more appropriate for comparing the relative trends in electron–phonon coupling strength among different samples or under different EL states. The associated uncertainties mainly arise from the fitting errors in peak position and peak area, the model dependence introduced by the selection of the three-peak fitting scheme and its constraints, the approximate nature of the construction of *ω_log_*, and the systematic uncertainty associated with the chosen value of the Coulomb pseudopotential μ*.

## 3. Results

**Figure 1:**
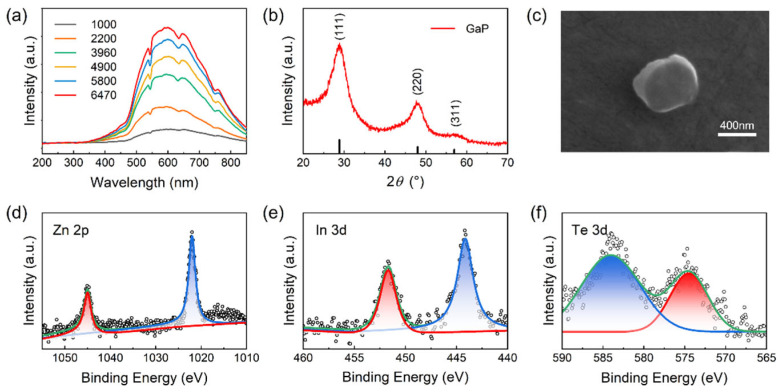
(**a**) Electroluminescence spectrum of the GaP electroluminescent inhomogeneous phase. (**b**) XRD pattern of the GaP electroluminescent inhomogeneous phase. (**c**) SEM image of the GaP electroluminescent inhomogeneous phase. (**d**–**f**) XPS spectra of the GaP electroluminescent inhomogeneous phase.

Figure 1 presents the structural characteristics and optical behavior of the GaP electroluminescent inhomogeneous phase after high-temperature treatment. The resulting particles comprise two types of multilayer core–shell units, namely [GaP:Zn/GaP]_×m_/[GaInP/GaP]_×n_ and [GaP:Te/GaP]_×m_/[GaInP/GaP]_×n_, which further assemble into nanoscale composite particles during synthesis. Figure 1a presents the electroluminescence spectra of different GaP inhomogeneous phases measured under the same current-bias condition. All samples exhibit a broad emission band spanning approximately 500–750 nm, with the emission center located near 600 nm, indicating their capability to provide a stable interfacial optical field within the visible spectral range. Morphological characterization (Figure 1c) shows that the particles form uniform spherical aggregates with a particle size distribution centered at approximately 400 nm, exhibiting good monodispersity. This high degree of size uniformity is important for maintaining the stability of the luminescence process. The XRD pattern (Figure 1b) displays characteristic zinc-blende GaP diffraction peaks at (111), (220), and (311), with no detectable impurity phases or extraneous reflections. This indicates that the dopant elements Zn, Te, and In are successfully incorporated into the GaP host lattice to form homogeneous solid solutions. The construction of the core–shell architecture does not induce any phase transformation, reflecting favorable lattice matching. The absence of noticeable peak shifts further suggests that heterovalent doping does not introduce measurable lattice distortion, which is crucial for preserving the electronic structure of the quantum-dot units. Complementary XPS measurements (Figure 1d–f) reveal well-defined characteristic peaks in the Zn 2p, Te 3d, and In 3d regions, demonstrating that the dopant elements are incorporated into the GaP lattice in chemically stable states, consistent with the XRD analysis.

**Figure 2:**
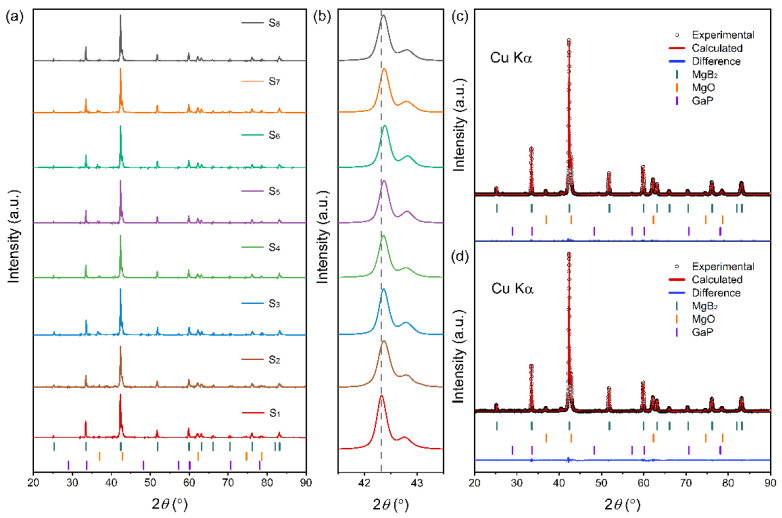
(**a**) X-ray diffraction (XRD) patterns of the pristine MgB_2_ sample (S_1_) and MgB_2_ composite samples containing 0.5 wt.% GaP electroluminescent inhomogeneous phases (S_2_–S_8_). (**b**) Enlarged view of the MgB_2_ (101) diffraction peak region. (**c**,**d**) Rietveld-refined XRD patterns of S_1_ and S_8_.

Figure 2 shows the X-ray diffraction (XRD) patterns of the pristine MgB_2_ sample (S_1_) and the MgB_2_ composite samples containing 0.5 wt.% GaP electroluminescent inhomogeneous phases (S_2_–S_8_). All samples exhibit the characteristic hexagonal MgB_2_ diffraction features, with only minor MgO impurity peaks (approximately 10–12 wt.%) detected in addition to the main reflections, indicating that the primary crystalline phase remains stable and that the incorporation of GaP does not alter the MgB_2_ host structure. No GaP-related diffraction peaks are observed, mainly because its concentration (0.5 wt.%) falls below the detection limit of XRD, combined with its small particle size and the partial overlap of its reflections with those of MgB_2_. Moreover, no new diffraction peaks associated with Ga- or P-containing secondary phases are detected, confirming that GaP retains its chemical and structural stability under the sintering conditions and persists as an intact inhomogeneous phase capable of forming well-defined GaP/MgB_2_ interfaces. It should be noted that MgO is commonly present in bulk MgB_2_, mainly originating from the surface oxidation layer of the MgB_2_ precursor powders and from residual O_2_/H_2_O during the sintering process. Even when weighing and mixing are performed in a glove box, it remains difficult to completely prevent ppm-level oxygen ingress during subsequent sealing and high-temperature treatment. For the composite samples, the surfaces of GaP particles may carry small amounts of oxides or organic residues; oxygen released from these species during the 850 °C heat treatment can further promote local MgO formation. Consequently, the MgO content obtained from Rietveld refinement is slightly higher in the composite samples (Table 1). It should be noted that MgO, as a common insulating secondary phase in the MgB_2_ system, may exert a certain influence on interparticle connectivity and intergrain current transport. However, as shown in Table 1, the MgO content in samples S_1_–S_8_ varies only within a relatively narrow range and does not exhibit a monotonic dependence on EL intensity. At the same time, its variation trend does not show a clear correspondence with either *T_c_* or the transition width. Therefore, MgO is better regarded as one of the background factors affecting connectivity and transport behavior, rather than the dominant origin of the systematic performance evolution discussed in this work.

Subtle shifts in the diffraction peaks reveal slight lattice modulation of MgB_2_ upon GaP incorporation. Rietveld refinement of the diffraction data (Table 1) shows that the a-axis lattice parameter increases marginally from 3.0853 Å to approximately 3.0883 Å, accompanied by a slight increase in the c-axis, resulting in a modest decrease in the c/a ratio. Such coexistence of in-plane expansion and mild interlayer contraction is typically associated with local strain fields introduced by nanoscale inhomogeneous phases during the sintering process, suggesting that the nanoscale dispersion of GaP within the matrix imposes a weak structural perturbation on the MgB_2_ lattice [37]. Notably, this minor evolution in lattice parameters does not compromise the crystallinity of the samples. The full width at half maximum (FWHM) of the (101) reflection exhibits only negligible variation, and the average crystallite size remains in the range of 0.11–0.12 μm, indicating that no significant structural defects or grain-boundary degradation are introduced in this composite system. These observations confirm that the GaP inhomogeneous phase does not disrupt the MgB_2_ host lattice or induce severe strain accumulation but instead modulates its microstructure in a mild and controlled manner.

**Figure 3:**
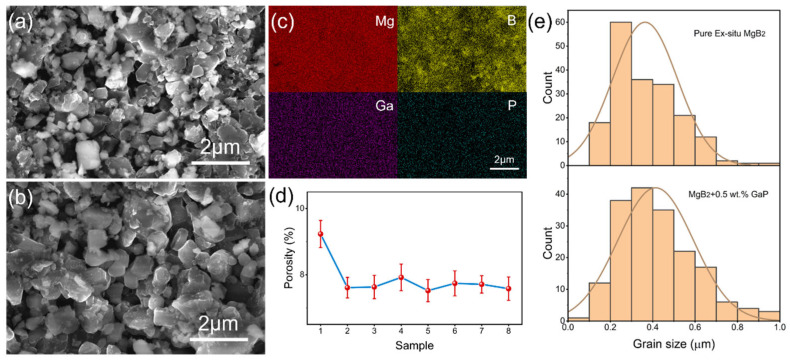
(**a**) SEM image of the pristine MgB_2_ sample S_1_. (**b**) SEM image of the composite sample containing 0.5 wt.% GaP (EL = 6600, S_8_). (**c**) EDS elemental mapping of the composite sample S_8_. (**d**) Porosity statistics of all samples. (**e**) Grain-size distribution histograms of the pristine sample S_1_ and the composite sample S_8_.

Figure 3 presents the microstructural morphology, elemental distribution, and porosity and grain-size statistics of the pristine MgB_2_ sample and the MgB_2_ sample incorporating 0.5 wt.% GaP electroluminescent inhomogeneous phases (EL = 6600). The pristine MgB_2_ sample (Figure 3a) exhibits a typical granular polycrystalline morphology in which the grains consist of agglomerated nanoscale primary particles forming a dense sub-grain network through intimate interparticle contacts. This microstructure ensures good connectivity of the matrix while still containing a limited amount of residual porosity. In contrast, the sample containing GaP inhomogeneous phases (Figure 3b) shows more fully developed grains with rounder particle morphology, tighter intergranular bonding, and noticeably larger grain sizes. These features suggest that the GaP electroluminescent particles function as heterogeneous nucleation sites for MgB_2_ growth, becoming encapsulated and embedded within the matrix and thereby promoting grain coarsening and grain-boundary densification during sintering. Because the GaP particles are uniformly distributed, their interfacial effects act cooperatively throughout the matrix, resulting in a more stable and compact grain-boundary framework that supports the formation of continuous current-carrying pathways.

The EDS elemental mapping of the 0.5 wt.% GaP (EL = 6600) sample (S_8_) is shown in Figure 3c. Mg and B elements are evenly distributed across the matrix, while the Ga and P signals appear as weakly dispersed features localized within nanoscale regions, with no evidence of aggregation or phase segregation. This observation further confirms that GaP inhomogeneous phases remain structurally intact and uniformly embedded at the nanoscale within the MgB_2_ matrix without decomposition. It should be noted that EDS elemental mapping typically employs a normalized color scale to display the relative count intensity of elements; therefore, even a small number of high-atomic-number elements (such as Ga) can exhibit relatively strong contrast. In addition, GaP tends to disperse preferentially along grain boundaries and pores. Since the two-dimensional elemental mapping covers a large grain-boundary network, relatively “widespread” Ga/P signals can be observed. Based on the nominal addition of 0.5 wt.%, the estimated volume fraction of GaP is only about ~0.3 vol.%, which is consistent with the absence of any obvious enrichment or agglomerated secondary phase. Figure 3d presents the porosity statistics of all samples, showing that the porosity decreases markedly after the addition of GaP electroluminescent particles, which indicates that the introduction of the inhomogeneous phase effectively improves the densification of the samples. This trend may be attributed to the role of GaP nanoparticles as “void fillers” during the sintering process, promoting intergrain contact and pore closure and thereby enhancing the overall compactness of the material. Grain-size statistics (Figure 3e) further elucidate their microstructural evolution. The pristine MgB_2_ sample exhibits a relatively broad grain-size distribution with an average size of 0.35 μm, whereas the GaP-containing sample shows a clear shift toward larger grain sizes with a narrower distribution and an increased average size of 0.45 μm. This indicates that the presence of the inhomogeneous phase moderately promotes grain growth by reducing interfacial energy at grain boundaries, which in turn helps decrease grain-boundary scattering and mitigates current-blocking effects. Combining the XRD and SEM analyses, it is evident that the GaP inhomogeneous phase, while preserving the MgB_2_ host crystal structure, effectively improves lattice connectivity by modulating microstructural morphology, porosity, and grain development.

**Figure 4:**
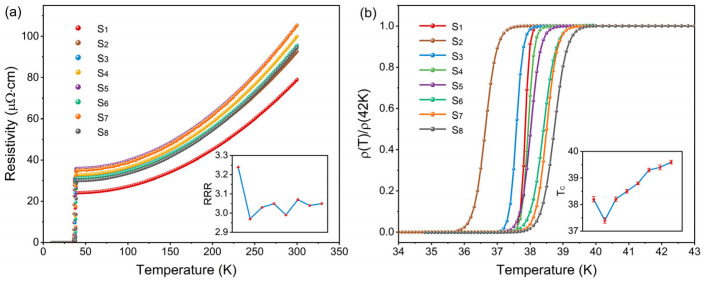
(**a**) Temperature-dependent resistivity curves (0–300 K) of pristine MgB_2_ and MgB_2_ composites containing 0.5 wt.% GaP electroluminescent inhomogeneous phases. Inset: variation of the residual resistivity ratio (*RRR*) with GaP emission intensity. (**b**) Enlarged view of the superconducting transition region. Inset: *T_c_* as a function of GaP emission intensity.

Figure 4 presents the temperature-dependent resistivity curves (0–300 K) and superconducting transition characteristics of the pristine MgB_2_ sample and the sample incorporating 0.5 wt.% GaP electroluminescent inhomogeneous phases. The pristine MgB_2_ sample exhibits a room-temperature resistivity *ρ*(300 K) of approximately 78 μΩ·cm, followed by a gradual decrease upon cooling and a sharp drop to zero near 38.2 K. This metallic behavior and abrupt superconducting transition indicate high crystallinity and excellent intergrain connectivity. In comparison, the sample containing 0.5 wt.% GaP inhomogeneous phases shows a slightly higher *ρ*(300 K). This increase arises primarily from the interfacial potential barriers and additional scattering centers formed between the GaP nanoparticles and the MgB_2_ matrix: after sintering, the uniformly distributed GaP particles reside preferentially at grain boundaries, and their electronic structure is not fully aligned with the metallic conduction band of MgB_2_. As a result, carriers experience barrier scattering and local electric-field perturbations when traversing these interfaces at room temperature, leading to an increase in *ρ*(300 K). Nevertheless, all composite samples preserve a clear metallic *ρ*-*T* trend, demonstrating that the macroscopic current-carrying pathways remain continuous and that GaP addition does not trigger non-metallic or localized transport behavior.

Despite the increase in *ρ*(300 K), the residual resistivity ratio (*RRR*) remains within a narrow range of 3.0–3.2 (Table 2), comparable to that of pristine MgB_2_. The stability of *RRR* implies that the intrinsic intragrain scattering mechanisms and electronic transport quality are preserved, and the primary modifications occur within the grain-boundary regions. To quantitatively evaluate the effective cross-sectional area of the macroscopic current paths, the Rowell connectivity analysis was employed:

$$∆\rho =\rho_{\left(300K\right)}-\rho_{\left(40K\right)}$$

$$A_{F}={\Delta \rho }_{ideal}/\Delta \rho$$

where Δ*ρ_ideal_* is taken as 7.3 μΩ·cm for an ideally dense MgB_2_ [38]. With the introduction of the GaP inhomogeneous phases, Δ*ρ* decreases slightly, leading to a small fluctuation of *A_F_* within the range of 0.103–0.115. The limited magnitude of this variation indicates that the effective conductive cross-section of the samples remains largely stable.

The superconducting transition provides further insight into the impact of GaP inhomogeneous phases on the pairing process. The pristine MgB_2_ sample exhibits a narrow transition width Δ*T_c_* ≈ 0.7 K, with a sharp and well-defined zero-resistance transition. For the sample S_2_ containing non-emissive GaP inhomogeneous phases, *T_c_*_,*onset*_ is slightly suppressed relative to the pristine sample, and the transition becomes marginally broader. This behavior agrees with prior reports on inert nanostructured inhomogeneous phases, in which interfacial scattering and local strain mildly hinder the pairing process [15,39,40,41]. As the GaP emission intensity increases from 1000 to 6600, the superconducting transition temperature *T_c_* rises steadily from 38.2 K to 39.6 K, while the zero-resistance temperature *T_c_*_,zero_ shifts upward synchronously and exceeds that of the pristine sample. This indicates that the entire transition region moves toward higher temperatures, rather than merely showing a local change in the onset transition temperature. Meanwhile, Δ*T_c_* exhibits only moderate variation, without any pronounced abnormal broadening or clear signatures of enhanced inhomogeneity. Considering the fixed GaP addition level, the non-emissive control sample, and the monotonic evolution of both *T_c_* and *T_c_*_,zero_ with EL intensity, the results suggest that, in addition to the structural perturbation introduced by static inhomogeneous-phase incorporation, the luminescence-activated state under bias excitation is likely involved in modulating the superconducting response [16,17,30,33].

Overall, the influence of GaP electroluminescent inhomogeneous phases on the electrical transport behavior exhibits multidimensional coupling effects: the increase in room-temperature resistivity is mainly associated with interfacial scattering, while the stability of *RRR* and *A_F_* suggests that the intragrain quality and the overall macroscopic connectivity remain generally well maintained. At the same time, the steady enhancement of *T_c_* with increasing EL intensity implies that, in addition to static structural effects, an additional modulation factor related to the luminescence-activated state may also be involved. Considering the non-emissive control sample and the fixed GaP addition level, this trend is consistent with an interpretation in which interfacial electromagnetic/optical activation effects participate in modifying the electron-pairing environment. Nevertheless, the present transport results alone do not allow the possible combined influence of local strain, defect scattering, grain-boundary effects, and other microstructural factors to be fully ruled out.

To ensure the reliability of the observed *T_c_* enhancement trend, key samples were independently re-fabricated and re-measured. The *T_c_*_,onset_ and *T_c_*_,zero_ values of the replicated samples were consistent with those of the primary experiments, with batch-to-batch variations within ±0.08 K, indicating good reproducibility of the *T_c_* evolution trend. In addition, the measurement uncertainty mainly arises from temperature control and electrode-contact factors, with an overall uncertainty estimated to be approximately ±0.07–0.10 K, which is substantially smaller than the systematic *T_c_* increase of about 1.4 K observed with increasing emission intensity.

A further point that merits discussion is that the bias current used in four-probe transport measurements may introduce Joule heating, contact heating, and local temperature gradients, thereby affecting the superconducting transition width and the apparent *T_c_*. Although a standard four-probe method was employed in this work, with alternating acquisition of voltage signals under positive and negative current directions to effectively eliminate thermoelectric offsets and significantly suppress background drift and low-frequency noise, thereby improving the stability of low-resistance measurements, this does not necessarily mean that microscopic local temperature rises within the sample, at the contact regions, or along local conduction paths can be completely excluded. At the same time, the temperature sensor was mounted on the back side of the sample stage in close contact with the sample, and the thickness of the stage was only about 1 mm. Its reading should therefore reflect the macroscopic temperature variation and temperature stability in the vicinity of the sample reasonably well, rather than merely the temperature of a remote low-temperature platform. It should be noted that these measures help reduce measurement artifacts arising from thermoelectric offsets and background drift, but they cannot be regarded as equivalent to completely excluding microscopic temperature rises within the sample, at the contact regions, or along local conduction paths. Nevertheless, in the present study, all samples were compared under the same bias current, the same measurement mode, and the same GaP addition level, and the sample resistance remained relatively low in the relevant temperature range. Therefore, the resulting Joule heating is expected to be limited and is unlikely, by itself, to account for the overall *T_c_* variation of approximately 1.4 K observed in this work. More importantly, at the same inhomogeneous-phase content, the non-emissive control sample did not exhibit the same enhancement trend as the emissive samples, while *T_c_* showed a systematic monotonic evolution with EL intensity, which differs markedly from the irregular shifts that might be expected if the behavior were dominated by random temperature drift or local-heating differences. A more cautious conclusion is therefore that, beyond static structural effects, there may also exist an additional interfacial activation effect related to electroluminescence under bias excitation. Although local thermal effects and nonequilibrium effects still cannot be completely excluded, they are unlikely to constitute, on their own, the sole origin of the systematic *T_c_* enhancement observed in this work. Their relative contributions still need to be further distinguished through more rigorous in situ local temperature measurements and control experiments in future studies.

**Figure 5:**
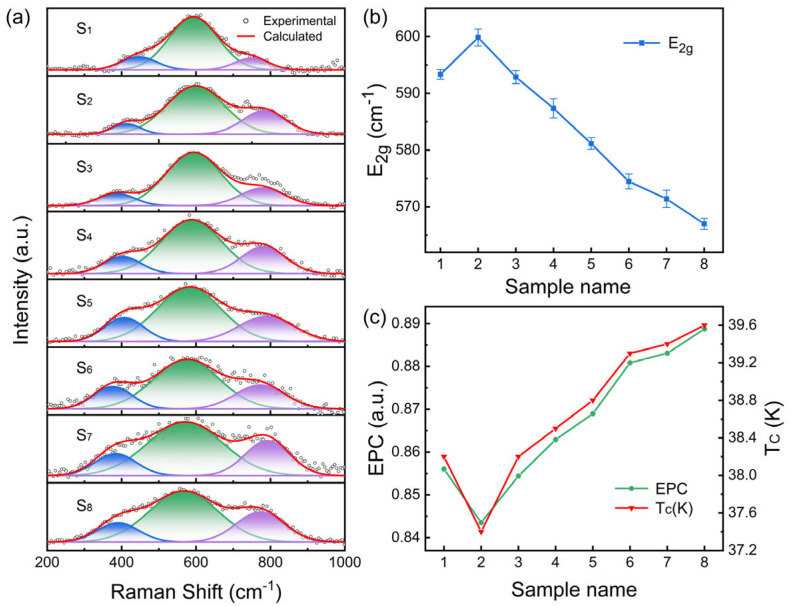
(**a**) Raman spectra and Gaussian fitting results of pristine MgB_2_ and MgB_2_ composites containing 0.5 wt.% GaP electroluminescent inhomogeneous phases under a 100 mA bias current, together with the corresponding three-peak Gaussian fitting results. The gray dots represent the experimental data, the red line represents the overall fitted curve, and the colored sub-peaks represent the three Gaussian components. (**b**) Systematic evolution of the *E_2g_* phonon frequency with increasing GaP emission intensity. (**c**) Concurrent evolution of *T_c_* and the semi-quantitatively estimated electron–phonon coupling parameter *λ* based on the Allen–Dynes relation.

Figure 5 shows the Raman spectra and corresponding three-peak Gaussian fitting results for the pristine MgB_2_ sample and the MgB_2_ composites containing 0.5 wt.% GaP electroluminescent inhomogeneous phases (EL = 1000–6600). All measurements were carried out under a 100 mA bias current to activate the GaP electroluminescent centers and generate interfacial electroluminescence and localized electromagnetic fields. The Raman spectra over the 200–1000 cm^−1^ range can be accurately decomposed into three Gaussian components. The broad mid-frequency peak corresponds to the in-plane B-B bond stretching vibration of the *E_2g_* phonon mode, the hallmark optical mode of MgB_2_ that is strongly linked to its electron–phonon coupling. The low-frequency peak mainly reflects acoustic-branch vibrations or defect-activated modes associated with grain boundaries, defects, and nanoparticles, whereas the high-frequency shoulder is generally attributed to second-order scattering, interface-related vibrational states, or locally disordered structures [8,42,43].

The evolution from pristine to composite samples can be divided into two distinct regimes. The first regime corresponds to the transition from S_1_ (pure MgB_2_) to S_2_ (MgB_2_ with non-emissive GaP particles). In this stage, the *E_2g_* peak undergoes a slight blue shift (hardening) accompanied by a minor linewidth adjustment, and the corresponding electron–phonon coupling constant *λ* decreases slightly. This indicates that when GaP acts solely as a “non-emissive inhomogeneous phase,” its primary effect is to introduce local strain and interfacial scattering, producing modest perturbations in the phonon potential landscape. These perturbations slightly increase the effective force constant of the *E_2g_* mode and consequently weaken the electron–phonon coupling. This interpretation is consistent with transport observations: *ρ*(300 K) increases whereas RRR remains nearly unchanged, reinforcing the view that non-emissive inhomogeneous phases act as “interfacial perturbations” rather than “pairing enhancers,” a behavior commonly reported in the literature [15,39,41,42,43,44].

The second regime occurs for composite samples with activated GaP electroluminescence (S_3_–S_8_). Upon increasing the electroluminescence intensity, the Raman *E_2g_* peak exhibits a systematic red shift and significant broadening. While the pristine MgB_2_ sample shows an *E_2g_* peak at 593 cm^−1^, the peak position progressively shifts to 570 cm^−1^ for the emissive composites, and the FWHM increases from 154 cm^−1^ to over 210 cm^−1^. The red shift of the *E_2g_* mode is generally associated with a softening of the B-B bond-stretching vibration, whereas the linewidth broadening is often related to an increase in phonon-scattering channels and a shortening of the phonon lifetime. At this stage, GaP no longer acts merely as a “static nanoscale phase,” but is activated as an electroluminescent center under the applied bias current. The interfacial activated state under bias excitation is therefore likely to participate in modulating the interaction environment between phonons and electrons, which is manifested in the vibrational response as the softening and broadening of the *E_2g_* mode [43].

To quantify the impact of this spectral evolution on superconducting pairing, the characteristic frequencies obtained from the three-peak fits were used to extract the logarithmic average phonon frequency. The electron–phonon coupling constant *λ* was then calculated using the Allen–Dynes method as described in the Experimental Section [45]. It should be emphasized that *λ* was indirectly extracted from the experimental *T_c_* and the fitted characteristic frequencies. Therefore, it is better regarded as an auxiliary parameter for comparing the relative evolution of coupling strength among different samples or under different EL states, rather than a high-precision absolute physical quantity. The results show that *λ* decreases slightly from S_1_ to the non-emissive S_2_, consistent with the modest *E_2g_* hardening. However, from S_3_ onward, *λ* increases systematically from 0.856 to 0.889 as the electroluminescence intensity rises, tightly correlating with the observed enhancement in *T_c_*. In other words, introducing GaP as a mere “static inhomogeneous phase” only introduces interfacial strain and scattering, leading to a slight reduction in *λ*; only when GaP is electrically excited—producing sustained interfacial optical fields and near-field electromagnetic modes—does *λ* increase beyond that of pristine MgB_2_, driving the monotonic rise in *T_c_*.

Taken together, the Raman spectra suggest that “non-emissive inhomogeneous phases” and “electroluminescent inhomogeneous phases” may correspond to two distinct modes of action in MgB_2_. The former is mainly manifested as local static strain and interfacial scattering effects, whereas the latter exhibits additional spectroscopic responses associated with an interfacial activated state under bias excitation. The slight blue shift of the *E_2g_* mode in S_2_, followed by the red shift and broadening observed in the emissive samples, together with the concurrent evolution of *λ* and *T_c_* with EL intensity, collectively support a possible physical picture in which the GaP electroluminescent inhomogeneous phase introduces an additional modulation channel at the interface beyond static structural perturbation. This channel may be related to local optical/electromagnetic near fields and may participate in regulating the key phonon modes and pairing environment of MgB_2_ [16].

It should be noted, however, that the red shift and linewidth broadening of the *E_2g_* mode are not unique signatures of interfacial light-field-phonon coupling. For a system such as MgB_2_, which is sensitive to local structure and scattering, local strain, defect introduction, enhanced grain-boundary disorder, shortened phonon lifetime, and possible local thermal effects under bias conditions may also give rise to similar Raman spectral evolution. Therefore, the observed *E_2g_* softening, FWHM increase, and the concurrent variation of *λ* and *T_c_* are more appropriately regarded as supportive evidence consistent with the proposed interfacial activation mechanism, rather than exclusive proof of it. At the same time, by fixing the GaP addition level, introducing a non-emissive control sample, and establishing a monotonic correlation among EL intensity, Raman features, and *T_c_*, the present study reduces, to some extent, the interpretive space for an explanation based solely on compositional variation, suggesting that the luminescence-activated state itself is likely involved in modulating the superconducting response. Nevertheless, to rigorously distinguish the relative contributions of interfacial near-field effects from those of local strain, defects, grain boundaries, and related factors, further verification will be required through more rigorous in situ temperature-controlled Raman measurements, local-field simulations, and additional control experiments.

**Figure 6:**
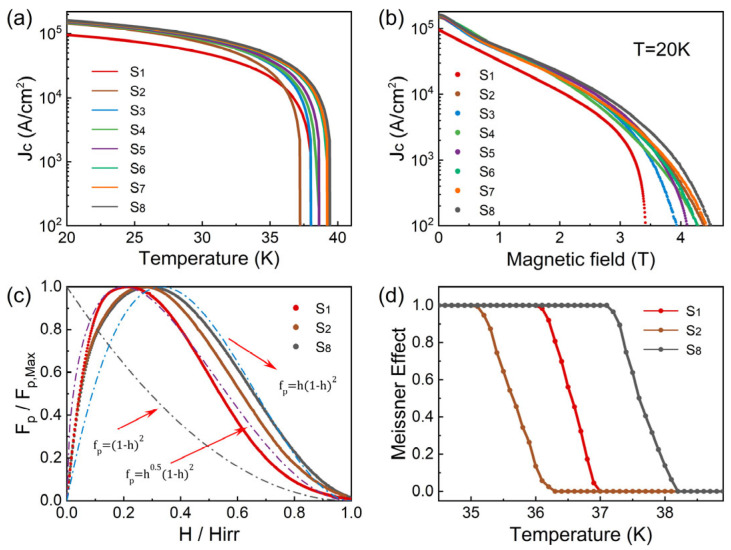
(**a**) *J_c_* − *T* curves of pristine MgB_2_ and MgB_2_ composites containing 0.5 wt.% GaP electroluminescent inhomogeneous phases. (**b**) *J_c_* − *H* curves. (**c**) *F_p_* − *H* pinning-force curves. (**d**) Meissner-effect curves, static field-repulsion strength estimated from levitation height.

Figure 6 presents the critical current density (*J_c_*), pinning force (*F_p_*), and Meissner effect for the pristine MgB_2_ sample and the sample incorporating 0.5 wt.% GaP electroluminescent inhomogeneous phases under various temperatures and magnetic fields. The *J_c_* − *T* curves (Figure 6a) show that under self-field conditions, the pristine MgB_2_ sample exhibits a *J_c_* of 9.55 × 10^4^ A·cm^−2^ at 20 K, whereas the composite sample reaches approximately 1.61 × 10^5^ A·cm^−2^, corresponding to a 69% enhancement. With increasing temperature, *J_c_* for both samples decays exponentially; however, the composite sample consistently maintains higher values across the entire temperature range. This indicates that the GaP electroluminescent inhomogeneous phase promotes sintering-induced densification, optimizes grain boundary structures, and introduces additional nanoscale dispersed phases/defects that act as effective pinning centers, thereby improving the current-carrying pathways and enhancing flux pinning capability.

The *J_c_* − *H* curves (Figure 6b) further reveal the pronounced high-field advantages introduced by GaP inhomogeneous phases. The pristine MgB_2_ sample shows a rapid reduction in *J_c_* with increasing external field, whereas the composite sample exhibits a notably slower decay. At 2 T, the pristine sample shows a *J_c_* of 1.11 × 10^4^ A·cm^−2^, while the composite reaches 2.18 × 10^4^ A·cm^−2^ (an increase of 97%). At 3 T, *J_c_* increases from 2.36 × 10^3^ A·cm^−2^ in the pristine sample to 6.46 × 10^3^ A·cm^−2^ in the composite, corresponding to an enhancement of 174%. The observed high-field enhancement of *J_c_* indicates that the GaP inhomogeneous phase forms new, nanoscale effective pinning centers within the MgB_2_ matrix, suppressing flux-line motion and enhancing flux pinning strength. Since the GaP particles are dispersed along grain boundaries and interface regions, the local strain fields and fluctuations in the mean free path they introduce act as randomly distributed point pinning sources, leading to a more gradual decay of the *J_c_* − *H* curves and an upward shift of *H_irr_*.

The pinning force *F_p_* − *H* curves (Figure 6c) exhibit a typical single-peak feature. For the pure sample, the peak position *h_m_* ≈ 0.218, close to the “surface pinning” scenario (*h_m_* ≈ 0.2) in the Dew-Hughes model, consistent with its small grain size and abundant grain boundaries. Upon GaP addition, *h_m_* shifts to ≈0.272, accompanied by a significant increase in *F_p,max_*, indicating that point-like pinning contributions introduced by the nanoscale inhomogeneous phase/defects effectively enhance the overall pinning, evolving the system from predominantly grain-boundary pinning to a mixed “grain-boundary + point pinning” regime. It is noteworthy that *h_m_* values in the range 0.2–0.3 generally indicate mixed pinning rather than a single mechanism: *h_m_* closer to 0.2 corresponds to surface-dominated pinning, while values approaching 0.33 reflect stronger point-like (*δl*-type) pinning. Therefore, the shift of *h_m_* from 0.218 to 0.272 can be interpreted as the GaP dispersed phase providing additional nanoscale condensation energy and mean free path fluctuations near grain boundaries, raising the flux depinning barriers and extending the effective pinning field range. This interpretation is consistent with the observed mid-to-high field enhancement in the *J_c_* − *H* curves.

Figure 6d shows the Meissner effect and the temperature-dependent variation in static field expulsion estimated from levitation height measurements [20]. The composite sample exhibits an earlier onset of diamagnetism, consistent with the elevated *T_c_*_,*onset*_ observed in resistivity measurements. Upon cooling, both samples achieve full diamagnetic screening, whereas the composite sample consistently displays stronger magnetic repulsion across the entire temperature range, indicating improved global shielding capability.

## 4. Discussion

Importantly, the enhancement trends reported here differ fundamentally from those obtained through conventional chemical doping of MgB_2_. Chemical doping typically relies on aliovalent substitution or interstitial incorporation, affecting electron–phonon coupling and vortex pinning by tuning carrier density, chemical pressure, or introducing impurity phases. However, such structural perturbations frequently induce lattice distortion, enhanced interfacial scattering, and grain-boundary degradation, which makes it difficult to achieve simultaneous improvement in *T_c_*, *J_c_*, and magnetic response. Enhancement of one property is often accompanied by the deterioration of another, which is a well-known limitation of most metal-doping and nanoparticle-addition strategies [46,47,48,49,50]. In contrast, the present study demonstrates that the GaP electroluminescent inhomogeneous phases improve *T_c_*, *J_c_*, and *H_irr_* concurrently as their emission intensity increases. The critical temperature increases by approximately 1.4 K, the self-field *J_c_* improves by nearly 70%, and *H_irr_* increases by over 30%. This “three-parameter co-enhancement” originates from an entirely different mechanism: rather than relying on static structural perturbations, the electroluminescent inhomogeneous phases generate interfacial optical fields and electromagnetic polarization modes under bias excitation, which couple to the *E_2g_* phonon mode and strengthen electron–phonon interactions without introducing additional scattering or grain-boundary degradation. Therefore, the “concurrent enhancement” observed in this work is not a mere repetition of conventional chemical doping effects; rather, it represents a parallel realization of near-field-activated pairing enhancement and structural pinning optimization within the same material system.

From the perspective of the underlying pathways, the performance enhancement observed in this work mainly arises from two types of contributions. The first is the microstructural optimization induced by the introduction of the GaP inhomogeneous phase, including improved densification, reduced porosity, and the formation of additional flux-pinning centers, which mainly benefits the enhancement of *J_c_*, *F_p_*, and H_irr_. The second is that the luminescent state of the GaP inhomogeneous phase corresponds systematically to the evolution of *T_c_* and the Raman features, suggesting that it is also likely involved in modulating the superconducting response. Therefore, the present work reflects a synergistic enhancement of superconducting performance arising from the combined effects of structural optimization and luminescence activation, rather than a simple repetition of conventional chemical-doping effects.

From the perspective of scalability, the strategy proposed in this work is not limited to the MgB_2_ system alone. In our previous studies, similar performance-enhancement phenomena were also achieved in a BSCCO matrix, indicating that the introduction of electroluminescent inhomogeneous phases and the use of interfacial activated states under bias excitation to regulate superconducting response may have a certain degree of cross-material applicability among different superconducting systems. On this basis, the present strategy may also be further extended to composite superconducting systems incorporating different types of luminescent inhomogeneous phases, different interfacial configurations, and different excitation modes.

## 5. Conclusions

This study systematically investigated the influence of GaP electroluminescent inhomogeneous phases on the superconducting properties of MgB_2_. The experimental results show that, at a fixed GaP addition level, *T_c_*, *J_c_*, and *H_irr_* of MgB_2_ all exhibit synergistic improvement with increasing emission intensity of the inhomogeneous phase. The superconducting transition temperature increases steadily from 38.2 K to 39.6 K, accompanied by a synchronous upward shift of the zero-resistance temperature. The self-field critical current density at 20 K increases by approximately 69%, while the enhancement reaches up to about 170% in the high-field range of 2–3 T; the irreversibility field *H_irr_* is improved by approximately 31.5%. These results indicate that, under the composite design and excitation conditions adopted in this work, the GaP inhomogeneous phase can enable the concurrent enhancement of *T_c_*, *J_c_*, and *H_irr_*. The above performance improvement is unlikely to arise from a single factor. On the one hand, the introduction of the GaP inhomogeneous phase contributes to the optimization of the sample microstructure and the enhancement of flux pinning, thereby promoting the improvement of *J_c_* and *H_irr_*. On the other hand, the coordinated evolution of *T_c_*, the Raman features, and the semi-quantitative *λ* parameter with EL intensity suggests that, beyond the effect of static inhomogeneous-phase incorporation, the luminescence-activated state under bias excitation is also likely involved in modulating the superconducting response.

It should be emphasized that the present experimental results are more appropriately regarded as supportive evidence consistent with the proposed interfacial activation mechanism, rather than direct proof of a specific light-field coupling pathway. Factors such as local strain, defect scattering, grain-boundary structure, and possible thermal effects under bias conditions may also jointly influence the evolution of the spectroscopic response and superconducting parameters. However, considering the systematic and monotonic dependence of *T_c_* on EL intensity observed in the emissive samples, together with the fact that the non-emissive control sample does not exhibit the same variation trend, thermal effects alone are unlikely to account for the *T_c_* enhancement and the related property changes observed in this work. Based on this understanding, future studies will further optimize the structural parameters of the inhomogeneous phase, expand the tunable range of EL intensity, and combine more rigorous in situ temperature-controlled characterization, local-field analysis, and control experiments to further distinguish the relative contributions of interfacial activation effects, thermal effects, and other possible factors, thereby establishing a clearer and more reliable intensity–response relationship.

## Figures and Tables

**Table 1:** Rietveld refinement parameters and superconducting properties of pristine MgB_2_ (S_1_) and MgB_2_ composite samples containing 0.5 wt.% GaP electroluminescent inhomogeneous phases (S_2_–S_8_).

X (wt.%)	EL Intensity	a (Å)	c (Å)	Crystallite Size (μm)	FWHM (101)	MgO (%)	*T_c_* (K)
0	0	3.0853 (3)	3.5234 (1)	0.109	0.214	10.4	38.2
0.5	0	3.0875 (1)	3.5265 (2)	0.119	0.228	11.5	37.4
0.5	1000	3.0883 (1)	3.5284 (2)	0.121	0.230	11.7	38.2
0.5	2200	3.0877 (1)	3.5268 (1)	0.118	0.227	12.1	38.5
0.5	3960	3.0871 (2)	3.5259 (2)	0.123	0.231	11.5	38.8
0.5	4900	3.0867 (1)	3.5263 (1)	0.117	0.228	12.0	39.3
0.5	5800	3.0885 (1)	3.5277 (1)	0.121	0.226	12.3	39.4
0.5	6600	3.0883 (1)	3.5272 (1)	0.112	0.225	11.6	39.6

**Table 2:** Superconducting transition temperatures and transport parameters of pristine MgB_2_ and MgB_2_ composites containing 0.5 wt.% GaP electroluminescent inhomogeneous phases.

Sample	EL Intensity	Doping Concentration (wt.%)	*T_c_* (K)	Δ*T_c_* (K)	δ*T_c_* (K)	*RRR*	*A_F_*
S_1_	0	0	38.2 ± 0.08	0.7	0	3.24	0.135
S_2_	0	0.5	37.4 ± 0.07	1.6	−0.8	2.98	0.104
S_3_	1000	0.5	38.2 ± 0.07	1.3	0	3.03	0.115
S_4_	2200	0.5	38.5 ± 0.05	1.2	0.3	3.05	0.109
S_5_	3960	0.5	38.8 ± 0.06	1.6	0.6	2.99	0.104
S_6_	4900	0.5	39.3 ± 0.07	1.8	1.1	3.06	0.113
S_7_	5800	0.5	39.4 ± 0.05	1.9	1.2	3.04	0.103
S_8_	6600	0.5	39.6 ± 0.07	1.8	1.4	3.05	0.115

## Data Availability

The original contributions presented in this study are included in the article. Further inquiries can be directed to the corresponding author.
